# Hyperoside Alleviates High Glucose-Induced Proliferation of Mesangial Cells through the Inhibition of the ERK/CREB/miRNA-34a Signaling Pathway

**DOI:** 10.1155/2020/1361924

**Published:** 2020-07-21

**Authors:** Le Zhang, Qian Dai, Lanlan Hu, Hua Yu, Jing Qiu, Jiyin Zhou, Min Long, Shiwen Zhou, Kebin Zhang

**Affiliations:** ^1^National Drug Clinical Trial Institution, Xinqiao Hospital, Army Medical University, Chongqing 400037, China; ^2^Center of Medical Experiment Technology, Xinqiao Hospital, Army Medical University, Chongqing 400037, China; ^3^Preventive Medicine Department, Xinqiao Hospital, Army Medical University, Chongqing 400037, China

## Abstract

**Purpose:**

Hyperoside, a flavonoid isolated from conventional medicinal herbs, has been demonstrated to exert a significant protective effect in diabetic nephropathy. This study aimed to determine the underlying mechanisms, by which hyperoside inhibits high glucose-(HG-) induced proliferation in mouse renal mesangial cells.

**Methods:**

Mouse glomerular mesangial cells line (SV40-MES13) was used to study the inhibitory effect of hyperoside on cell proliferation induced by 30 mM glucose, which was used to simulate a diabetic condition. Viable cell count was assessed using the Cell Counting Kit-8 and by the 5-ethynyl-20-deoxyuridine incorporation assay. The underlying mechanism involving miRNA-34a was further investigated by quantitative RT-PCR and transfection with miRNA-34a agomir. The phosphorylation levels of extracellular signal-regulated kinases (ERKs) and cAMP-response element-binding protein (CREB) were measured by Western blotting. The binding region and the critical binding sites of CREB in the miRNA-34a promoter were investigated by the chromatin immunoprecipitation assay and luciferase reporter assay, respectively.

**Results:**

We found that hyperoside could significantly decrease HG-induced proliferation of SV40-MES13 cells in a dose-dependent manner, without causing obvious cell death. In addition, hyperoside inhibited the activation of ERK pathway and phosphorylation of its downstream transcriptional factor CREB, as well as the miRNA-34a expression. We further confirmed that CREB-mediated regulation of miRNA-34a is dependent on the direct binding to specific sites in the promoter region of miRNA-34a.

**Conclusion:**

Our cumulative results suggested that hyperoside inhibits the proliferation of SV40-MES13 cells through the suppression of the ERK/CREB/miRNA-34a signaling pathway, which provides new insight to the current investigation on therapeutic strategies for diabetic nephropathy.

## 1. Introduction

Diabetic nephropathy (DN) is a common microvascular complication of diabetes mellitus that is considered as one of the main causes of end-stage renal diseases. Glomerular hypertrophy, mesangial expansion, and a thickened glomerular basement membrane all result in glomerulosclerosis, which is one of the prominent characteristics of glomerular pathology in this disease [1]. Accumulating evidence suggests that the increased proliferation of glomerular mesangial cells is critical for the initiation and progression of DN [2, 3]. Mesangial cell proliferation is stimulated at the early-stage of DN; subsequently, the cell growth is arrested, and the cells undergo hypertrophy [4]. Therefore, inhibition of proliferation of mesangial cells may be an appropriate therapeutic approach to control DN progression at its initial stages.

Hyperoside (quercetin-3-O-*β*-D-galactopyranoside) is a natural compound that is extracted from the fruits and whole grasses of plants such as *Hypericaceae* and *Rosaceae*. Hyperoside has been shown to exert diverse pharmacological activities, including anti-inflammatory, antioxidation, antihyperglycemic, and cardiovascular protective properties [5–7]. In addition, hyperoside is the main active ingredient of Huangkui capsule, which is widely used for the clinical treatment of DN, chronic nephritis, and other renal diseases [8]. A recent study suggested that hyperoside could prevent glomerular basement membrane damage by decreasing the expression of podocyte heparanase in DN mice [9]. Moreover, our previous study also demonstrated that hyperoside has protective effects on kidney injury in mice with DN, which were achieved through ameliorating mesangial expansion and renal fibrosis [10]. However, its protective role and the underlying mechanisms of high glucose-(HG-) induced proliferation of mesangial cells in DN remain to be fully elucidated.

MicroRNA-34a (miR-34a) is a small noncoding RNA that negatively regulates the coding mRNAs at the posttranscriptional level and plays important roles in several biological processes. Numerous studies have revealed that miR-34a have a significant impact in the pathogenesis of diabetes and its related complications, including DN. miR-34a was overexpressed in type 2 diabetes mellitus and DN patients and exerted a regulatory effect on the urinary albumin creatinine rates, endothelial dysfunction, or mesangial cells proliferation and fibrosis caused by DN through the regulation of the expression of multiple target genes [11, 12]. By directly targeting growth arrest specific 1, the downregulation of miR-34a inhibited mesangial proliferation and glomerular hypertrophy in early DN [13].

It is well known that the extracellular signal-regulated kinase (ERK) signaling pathway is involved in the regulation of numerous important biological processes, including the biogenesis of miRNAs at the cellular level [14]. ERK1 and ERK2, involved in the ERK signaling pathway, are members of the mitogen-activated protein kinase (MAPK) family and are also involved in various physiological processes of cells, such as in the regulation of cell growth, division, and death. Activated ERK can promote nuclear transport of its downstream transcription factors such as c-Myc, c-Ets, and CREB, which in turn improves the transcription of the corresponding target genes [15, 16]. A previous study demonstrated that hyperoside could prevent HG-induced epithelial-mesenchymal transition via the inhibition of ERK1/2 activation in human kidney proximal tubule epithelial cells [17].

This study aimed to elucidate the underlying mechanisms, by which hyperoside inhibits HG-induced proliferation of mouse renal mesangial cells. Our results showed that hyperoside attenuated HG-induced proliferation of mesangial cells through the inactivation of the ERK/CREB/miR-34a signaling pathway. These findings are expected to provide new insights to drug development strategies for DN in the future.

## 2. Materials and Methods

### 2.1. Cell Culture and Treatment

Mouse renal mesangial cells (SV40-MES13) were purchased from Shanghai Cell Bank of Chinese Academy of Science, and cultured in DMEM/F12 medium (Gibco, U.S.A.) containing 5% fetal bovine serum (FBS, Gibco, U.S.A.). For the low-glucose group, cells were stimulated with 5.5 mM D-glucose, together with 24.5 mM mannitol; and for the high-glucose group, cells were stimulated with 30 mM glucose. Cells were incubated in humidified 95% air with 5% carbon dioxide at 37°C.

### 2.2. Cell Transfection

miRNA antagomir or agomir is a type of specially labeled and chemically modified miRNA. Antagomir is special for inhibiting the expression of endogenous miRNA. Agomir can regulate the biological function of target gene by mimicking endogenous miRNA. Antagomir-34a, agomir-34a, or matched negative controls (antagomir-NC, agomir-NC) was purchased from GenePharma (Shanghai, China). CREB short hairpin RNA (CREB-shRNA) (Oligobio, Beijing, China) was constructed to specifically block CREB according to the manufacturer's instructions. The cDNA sequences encoding CREB protein were subcloned into the pcDNA3.1-HA vector (Youbio, Hunan, China) to generate the CREB expression plasmid. Cell transfection was performed with Lipofectamine 2000 Reagent (Invitrogen, USA), as per the manufacturer's manuals.

### 2.3. Cell Counting Kit-8 (CCK-8)

Viable cell count was assayed by a colorimetric procedure, using CCK-8 (Dojindo, Shanghai, China) according to the manufacturer′s protocol. Briefly, 10 *μ*l of the CCK-8 solution was added to each well and incubated 2 h in a humidified incubator. The optical density (OD) values at 450 nm were measured using a microplate reader (Varioskan Flash, Thermo Scientific, USA).

### 2.4. 5-Ethynyl-20-Deoxyuridine (EdU) Incorporation Assay

An EdU incorporation assay was performed with the Cell-Light™ EdU In Vitro Imaging Kit (RiboBio, Guangzhou, China) to detect the proliferation rates of SV40-MES13 cell. Briefly, cells subjected to different treatment were incubated with 10 *μ*M EdU in complete growth medium for 2 h before fixation in 4% paraformaldehyde. After EdU staining, cell nuclei were stained with Hoechst 33342 and visualized under fluorescence microscopy (Leica, Germany). The proliferation rate was calculated by the number of EdU-stained cells normalized by the number of Hoechst 33342-stained cells.

### 2.5. Cell Apoptosis Analysis

After cells were exposed to the indicated concentration of hyperoside for 48 h, cell apoptosis was quantified by flow cytometry with an PE-Annexin V/7AAD apoptosis detection assay according to the manufacturer's protocol (BD Biosciences).

### 2.6. Real-Time Quantitative PCR (qRT-PCR)

Total RNA was isolated using TRIzol reagent (Invitrogen) according to manufacturer's instructions and reverse-transcribed into cDNA using a PrimeScript RT reagent Kit (TaKaRa, Tokyo, Japan). miRNA qRT-PCR was performed using the SYBR Premix Ex Taq kit (Takara, Tokyo, Japan) and an Applied Biosystems 7500 Fast real-time PCR system. The stem-loop primers used for the PCR amplification were synthesized by RiboBio (Guangzhou, China). All reactions were run in triplicate. The relative expression level of miR-34a was normalized against the U6 expression level. Fold changes relative to control samples were determined by the 2^−^^ΔΔ^Ct^^ method.

### 2.7. Western Blot Analysis

Total protein was extracted with RIPA buffer (Beyotime, Jiangsu, China), according to the manufacturer's instructions. Protein samples were subjected to SDS-PAGE and then transferred to polyvinylidene difluoride membranes (Millipore, Billerica, USA). Membranes were blocked with 5% nonfat dried milk at room temperature for 1 h and incubated with primary antibodies against phospho-CREB, CREB, phospho-ERK, ERK (1 : 1,000; CST, Cambridge, USA), and *β*-actin (Bioss, Beijing, China) overnight at 4°C. After being rinsed with Tris-buffered saline Tween-20, the membranes were incubated with secondary antibody for 1 h at room temperature. Protein-antibody complexes were visualized by an enhanced chemiluminescence kit (Thermo Scientific, USA).

### 2.8. Chromatin Immunoprecipitation (ChIP) Assay

ChIP assays were carried out in SV40-MES13 cell with a ChIP kit according to the manufacturer's instructions (Thermo Fisher Scientific, Waltham, MA). Briefly, cells were formaldehyde cross-linked. Cross-linked chromatin was sheared by ultrasonic crushing. The purified chromatin was immunoprecipitated with anti-CREB antibody or normal IgG antibody (CST, Cambridge, USA). The DNA/protein complexes were then collected by using Protein G-Agarose beads. After the reverse cross-linking reaction, the eluted DNA was used in 35 cycles of PCR amplification with primers designed for miR-34a promoter regions (forward: GGTACCTACCCCCTTTCTAAGACA, reverse: CTCGAGCACGTGGGGTCATTTCCA).

### 2.9. Luciferase Reporter Assay

The following sequences are predicted and synthesized: (1) wild-type miR-34a promoter sequence containing predicted CREB-binding site 1, 2, and 3; (2) mutant miR-34a promoter sequence deleting the binding site 1; (3) mutant miR-34a promoter sequence deleting the binding site 2; and (4) mutant miR-34a promoter sequence deleting the binding site 3. The four kinds of promoter sequences were constructed into the pGL3-basic vector, respectively, and a series of promoter constructs including wild-type (WT), mutant 1 (Mut1), Mut2, and Mut3 were generated.

293T cells were cotransfected with a reporter plasmid and pcDNA3.1-HA-CREB expression plasmid or empty vector. The luciferase activities were measured in a luminometer with the dual-luciferase reporter assay system (Promega, Madison, USA) according to the manufacturer's recommendations.

### 2.10. Statistical Analysis

All data are presented as mean ± SD. Differences between experimental groups were evaluated using the independent samples t-test or a one-way ANOVA followed by Tukey's multiple-comparisons test with SPSS 18.0 package (SPSS Inc., Chicago, Ill., USA). A *P* value < 0.05 was considered to be statistically significant.

## 3. Results

### 3.1. Hyperoside Inhibits the Proliferation of SV40-MES13 Cells by Downregulating miR-34a under HG Condition

**Figure 1:**
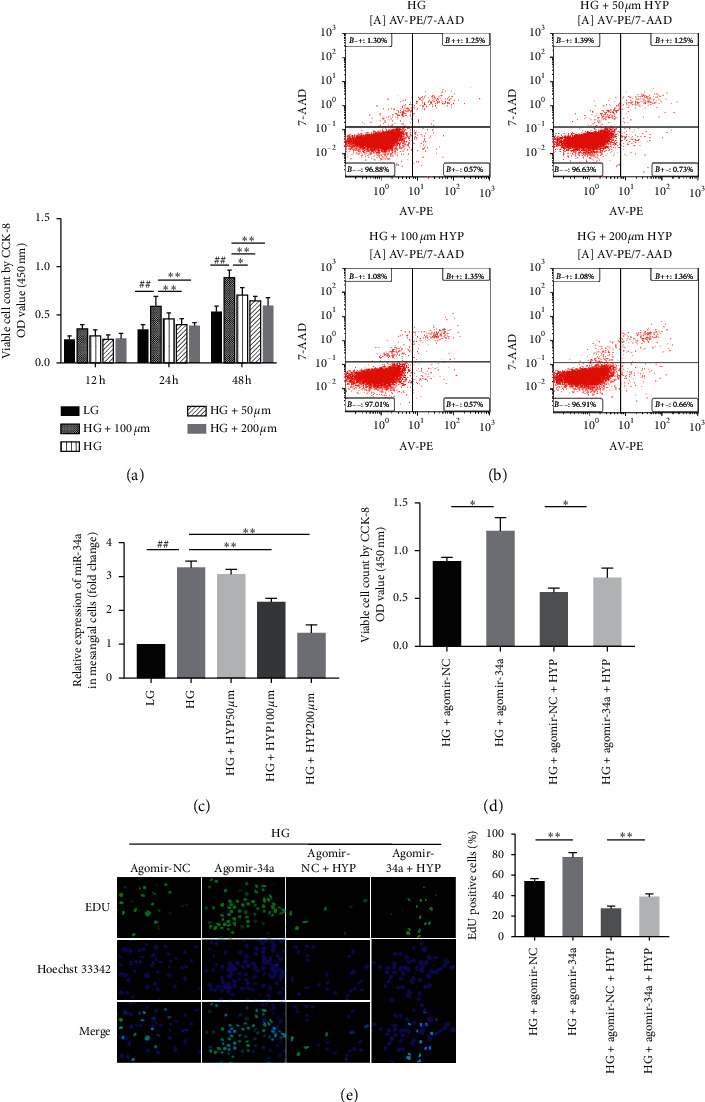
Hyperoside inhibits HG-induced SV40-MES13 cells proliferation by downregulating miR-34a. (a) Time-dependent effect and dose-dependent (50, 100, 200 *μ*M) effect of hyperoside on viable cell count of SV40-MES13 cells, as determined by CCK-8. (b) Cell apoptosis were analyzed by flow cytometry with the PE-Annexin V/7AAD staining. Representative data from triplicate experiments are shown. (c) SV40-MES13 cells were treated with LG or HG with different concentration of hyperoside for 48 h. The miR-34a expression was determined by qRT-PCR. (d) SV40-MES13 cells were transfected with agomir-34a or agomir-NC and then cultured with HG for 48 h with or without 200 *μ*M hyperoside. Cell proliferation was determined by CCK-8. (e) Cell proliferation rates were detected by EdU incorporation and the quantitative analysis (##*P* < 0.01; ^*∗*^*P* < 0.05, ^*∗∗*^*P* < 0.01). LG: low glucose (5.5 mM); HG : high glucose (30 mM); and HYP: hyperoside.

To validate the protective effect of hyperoside, the viable cell count of SV40-MES13 cells induced by HG was determined by the CCK-8 assay. We found that the proliferation of SV40 MES-13 cells was increased in a time-dependent manner by HG induction. After treatment with hyperoside, the cell proliferation was gradually inhibited by an increasing concentration of hyperoside, particularly at the concentration of 200 *μ*M (Figure 1(a)). Moreover, we determined the effect of hyperoside on the induction of SV40-MES13 cells apoptosis by flow cytometry with Annexin V-PE/7AAD staining. Our results revealed that hyperoside treatment did not increase apoptosis in SV40-MES13 cells (Figure 1(b)).

Accordingly, we next explored the role of miR-34a in the inhibitory effect of hyperoside against cell proliferation stimulated by HG. The qRT-PCR results demonstrated that miR-34a was significantly upregulated in HG-induced SV40-MES13 cells, whereas hyperoside decreased the expression of miR-34a in a dose-dependent manner (Figure 1(c)). The exogenous miR-34a expression by agomir transfection blocked the inhibitory effect of hyperoside on cell proliferation (Figures 1(d) and 1(e)). Together, these results indicate that hyperoside suppressed the proliferation of SV40-MES13 cells through miR-34a under HG condition.

### 3.2. Hyperoside Suppresses the miR-34a Expression via the Inhibition of ERK Activation

**Figure 2:**
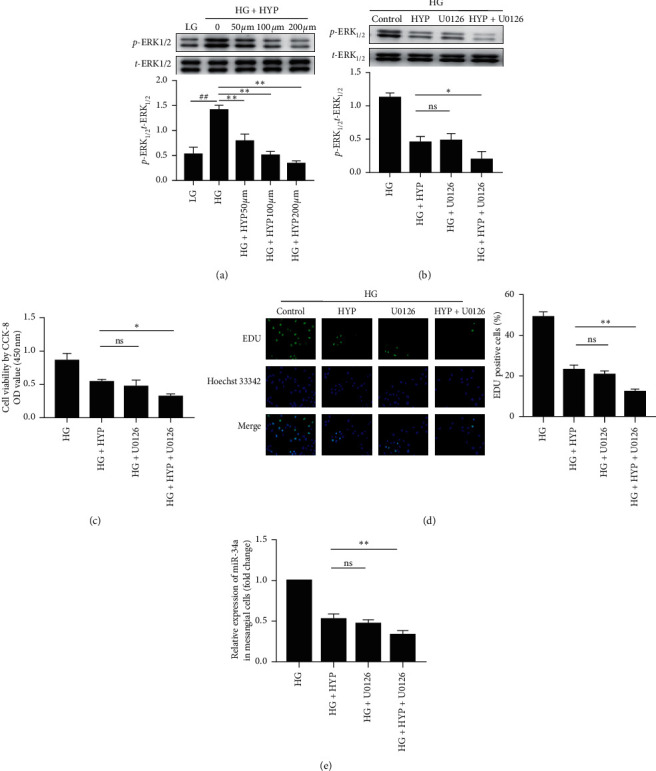
Hyperoside suppresses miR-34a expression via the inhibition of ERK activation. (a) SV40-MES13 cells were treated with LG or HG with different concentration of hyperoside for 48 h. The phosphorylation level of ERK was examined by Western blot. (b) SV40-MES13 cells treated with or without U0126 (10 *μ*M) were concurrently treated with or without hyperoside (200 *μ*M) for 48 h under HG condition. The phosphorylation level of ERK was determined by Western blotting. ((c), (d)) Cell proliferation was determined by CCK-8 and the EdU incorporation assay. (e) The expression of miR-34a was detected by qRT-PCR (##*P* < 0.01, ^*∗*^*P* < 0.05, ^*∗∗*^*P* < 0.01, n.s. indicates no significance).

To address whether the inhibitory effect of hyperoside on cell proliferation is associated with the ERK pathway, we estimated the phosphorylation level of ERK. We found that HG led to an increased phosphorylation level of ERK in SV40-MES13 cells. This observation suggested that the ERK activation was related to HG-induced cell proliferation, and hence, the inhibition of ERK activation may reduce cell proliferation. In accordance with this speculation, hyperoside treatment was found to significantly inhibit the phosphorylation level of ERK in SV40-MES13 cells (Figure 2(a)).

To further verify these results, the SV40-MES13 cells stimulated with HG were treated with the ERK inhibitor U0126, and the phosphorylation levels of ERK, cell proliferation, and miR-34a expression were then analyzed. The cells treated with U0126 showed reduced ERK phosphorylation level (Figure 2(b)) as well as reduced cell proliferation (Figures 2(c) and 2(d)) and miR-34a expression (Figure 2(e)). No significant differences were recorded in the ERK phosphorylation level, cell proliferation, or miR-34a expression between the U0126 and hyperoside treatment groups.

### 3.3. Hyperoside Regulates miR-34a through the ERK/CREB Pathway

**Figure 3:**
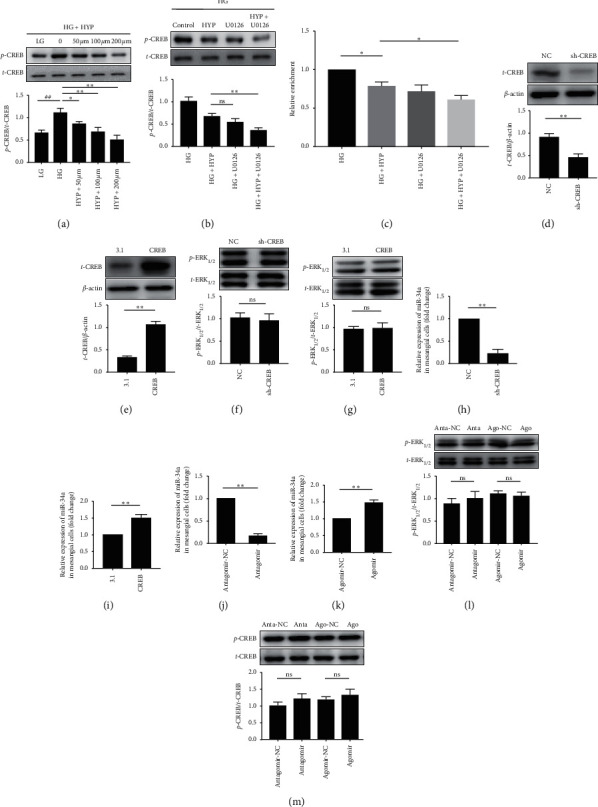
Hyperoside regulates miR-34a through the ERK/CREB pathway. (a) SV40-MES13 cells were treated with LG or HG with different concentrations of hyperoside for 48 h. The phosphorylated CREB was examined using Western blot. (b) SV40-MES13 cells treated with or without U0126 (10 *μ*M) were concurrently treated with or without hyperoside (200 *μ*M) for 48 h under HG condition. The phosphorylation level of CREB was examined by Western blot. (c) SV40-MES13 cells were treated or untreated with hyperoside (200 *μ*M) in the absence or presence of U0126 (10 *μ*M). The qRT-PCR analysis of sheared DNA from control and hyperoside-treated cells after ChIP with antibody directed against CREB. (d–g) Protein expression of CREB and the phosphorylation level of ERK in CREB-shRNA or pcDNA3.1-HA-CREB expression plasmids transfected cells were detected by Western blot, and (h, i) the expression of miR-34a was detected by qRT-PCR. (j, k) The expression of miR-34a in antagomir-34a or agomir-34a transfected cells was detected by qRT-PCR, and (l, m) the phosphorylation levels of ERK and CREB were detected by Western blot (##*P* < 0.01, ^*∗*^*P* < 0.05, ^*∗∗*^*P* < 0.01, n.s. indicates no significance).

The promoter of miR-34a possesses CREB-binding sites, and the CREB expression is regulated by ERK activation [18, 19]. Therefore, we investigated whether CREB was involved in the regulation of miR-34a by hyperoside. As shown in Figure 3(a), HG was observed to significantly induce the expression of phosphorylated CREB in SV40-MES13 cells, while hyperoside was noted to reduce phosphorylation of HG-stimulated CREB in a dose-dependent manner (Figure 3(a)). We also observed that the phosphorylation level of CREB was downregulated by treatment with U0126 (Figure 3(b)). To determine whether hyperoside regulate the binding of CREB to the promoter region of miR-34a, we performed the ChIP assay. As shown in Figure 3(c), hyperoside significantly decreased the binding of CREB to the miR-34a promoter.

We further confirmed the upstream and downstream relationships among ERK, CREB, and miR-34a. For this purpose, we first successfully interfered and overexpressed CREB expression by transfecting CREB shRNA and overexpression plasmid into SV40-MES13 cells, respectively (Figures 3(d) and 3(e)). We found that silencing and overexpressing CREB had no effect on ERK phosphorylation (Figures 3(f) and 3(g)), albeit a positive regulatory effect on the miR-34a expression (Figures 3(h) and 3(i)). These results suggested that CREB is a downstream target of the ERK pathway and possibly an upstream regulator to miR-34a. Moreover, we transfected agomir-34a or antagomir-34a into SV40-MES13 cells to confirm the effect of miR-34a on the ERK and CREB expressions. With the transfection of agomir-34a into SV40-MES13 cells, the miR-34a expression was significantly increased, while the expression of miR-34a was reduced by the treatment with antagomir-34a (Figures 3(j) and 3(k)). The upregulation or downregulation of miR-34a had no effect on the phosphorylation levels of ERK and CREB (Figures 3(l) and 3(m)). Collectively, we noted that hyperoside inhibited the expression of miR-34a in SV40-MES13 cells via the effect of the ERK/CREB pathway.

### 3.4. CREB-Mediated Transcriptional Regulation of miR-34a

**Figure 4:**
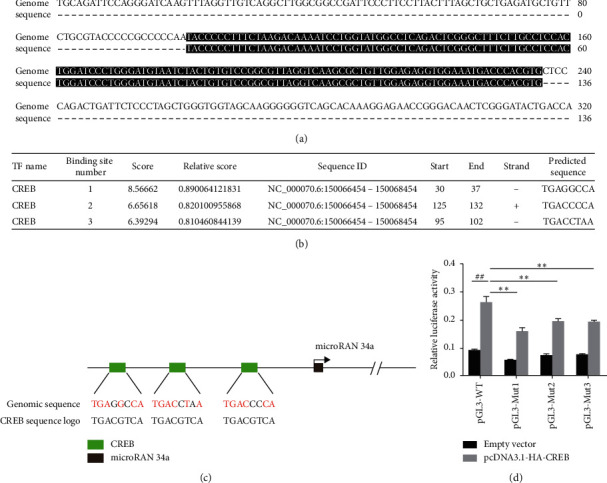
CREB-mediated transcriptional regulation of miR-34a. (a) Binding region sequence between CREB and miR-34a promoter was obtained by the ChIP assay and DNA sequencing. (b) Binding sites of CREB in the miR-34a promoter region were predicted according to an online prediction. (c) Schematic diagram of the putative CREB-binding sites in the promoter region of miR-34a. (d) Relative activities of luciferase reporters carrying the binding sites of CREB in the miR-34a promoter region that were cotransfected with either pcDNA3.1-HA-CREB or pcDNA3.1 empty vector into 293T cells.

To determine whether the transcription factor CREB may bind to the promoter of miR-34a, the ChIP assay was performed. DNA sequencing helped to identify the binding region between CREB and miR-34a promoter (Figure 4(a)). Next, we found 3 predicted critical binding sites of CREB in the miR-34a promoter region as per online prediction (http://jaspar.genereg.net/) (Figures 4(b) and 4(c)). These results showed that CREB-mediated transcriptional regulation of miR-34a is dependent on the direct binding of CREB to specific sites on the promoter region of miR-34a.

To identify the critical binding sites involved in the CREB-mediated regulation of miR-34a, we constructed miR-34a promoter luciferase reporters containing wild-type or mutant CREB-binding sites and conducted the luciferase reporter assay. As shown in Figure 4(d), the luciferase activity of CREB-overexpressed cells was significantly higher as compared to that of empty vector transfected cells, which indicates that CREB was essential for the miR-34a promoter activation. In comparison with the WT promoter construct, the luciferase activities of Mut1, Mut2, and Mut3 were significantly reduced. This finding suggested that the critical sites of CREB-mediated regulation of miR-34a were TGAGGCCA, TGACCCCA, and TGACCTAA.

## 4. Discussion

Mesangial cells play a key role in influencing the pathological changes in DN. In fact, mesangial expansion caused as a result of cell hyperproliferation decreases the glomerular filtration rate and occurs in patients with DN before the onset of clinical manifestations [20]. Therefore, inhibiting the proliferation of mesangial cells has become an important approach in the prevention and treatment of DN. Hyperglycemia is considered to be the initiation factor and the central links between renal hypertrophy and diabetes [21]. In this study, the proliferation of mesangial cells was significantly increased by stimulation with HG for 24 h and 48 h, as also reported elsewhere [22].

Hyperoside has been reported to exhibit anticancer activities by inhibiting cell proliferation and causing cell cycle arrest in several cancer cell lines [23–25]. In our study, we also noted that cell proliferation was markedly decreased in hyperoside-treated cells under HG condition. Furthermore, we demonstrated that miR-34a agomir could partially reverse the inhibitory effect of hyperoside on cell proliferation, which suggested that hyperoside could suppress the proliferation of mesangial cell partially by downregulating the expression of miR-34a.

ERK signaling is an important intracellular signal transduction pathway that contributes to the proliferation of mesangial cells, with several studies having demonstrated that specific inhibitors of the ERK pathway can prevent HG-induced cell proliferation in glomerular mesangial cells [26, 27]. Furthermore, the ERK pathway also participates in the biogenesis of miRNAs at the cellular level [14]. In this study, we found that hyperoside inhibited HG-induced cell proliferation and miR-34a expression by suppressing the ERK pathway. In addition, on the basis of hyperoside treatment, further combination with ERK inhibitor U0126 resulted in synergistic inhibition.

To further analyze how the ERK pathway regulates the miR-34a expression in HG condition, we conducted a bioinformatics prediction assay to determine the possible transcription factor binding site sequences of the miR-34a promoter region according to an online prediction software. We found putative CREB-binding sites located at the proximal promoter of miR-34a. Therefore, we speculated that CREB was regulated by ERK. Other past studies [18, 19] in conjunction with our findings thus indicate that CREB is located downstream of the ERK.

As a transcriptional activator, CREB generally binds to the CREB-binding protein (CBP) of the regulatory region of the target gene elements that promote the transcription of the target genes [28]. The targets of CREB are involved in regulating the normal renal physiological processes such as cell proliferation, differentiation, and apoptosis. CREB also plays an important transduction role in cell and intermolecular signaling pathways of the kidney in DN [29, 30]. In addition to protein-coding genes, noncoding targets, including miRNAs, can also be directly regulated by CREB. Recently, some miRNAs targets of CREB were identified during gliomagenesis and in Japanese encephalitis virus infection [15, 31]. Fortunately, we identified the occurrence of direct binding between CREB and miR-34a promoter regions at the binding sites TGAGGCCA, TGACCCCA, and TGACCTAA.

**Figure 5:**
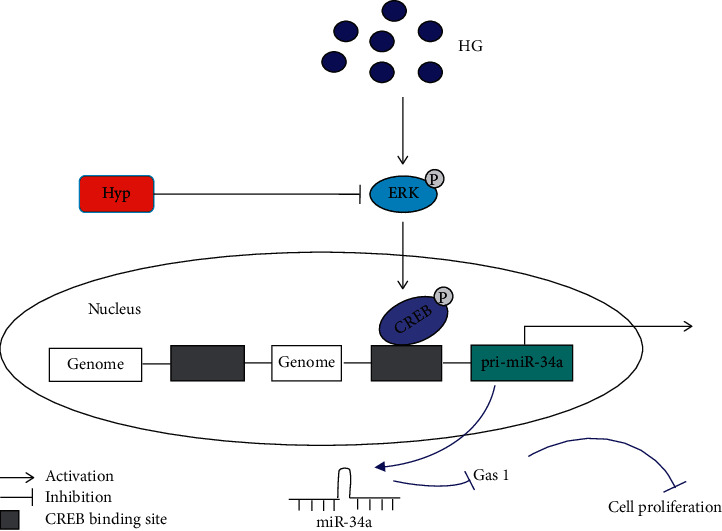
Schematic of the mechanism by which hyperoside inhibits the proliferation of mesangial cells in diabetic nephropathy model.

In summary, our study findings revealed that hyperoside inhibits the proliferation of mesangial cells by regulating the miR-34a expression through the ERK pathway, and that CREB-mediated transcriptional regulation of miR-34a is dependent on the direct binding of CREB to the miR-34a promoter (Figure 5). We believe that the ERK/CREB/miR-34a signaling pathway contributes to glomerular hyperproliferation during the progression of DN, and the strategies regulating this pathway may offer potential target for DN therapy. Thus, we clarified the new pathogenesis of DN as well as provided a scientific basis for the development and utilization of hyperoside through our present findings, which are expected to provide useful insights to the drug development field in DN.

## Data Availability

The data used to support the findings of this study are available from the corresponding author upon request.
